# Performance, carcass characteristics and non-carcass components of Santa Ines and crossbred (Santa Ines x Dorper) lambs finished in different confinement strategies

**DOI:** 10.1371/journal.pone.0293819

**Published:** 2023-11-09

**Authors:** Alinne Andrade Pereira, Luciara Celi Chaves Daher, Carolina Sarmanho Freitas, Samanta do Nascimento Monteiro, Jonas Carneiro Araújo, Marco Antônio Paula de Sousa, Andrey de Sousa Miranda, Thomaz Cyro Guimarães de Carvalho Rodrigues, Jamile Andrea Rodrigues da Silva, Alyne Cristina Sodré de Lima, André Guimarães Maciel e Silva, José de Brito Lourenço-Júnior

**Affiliations:** 1 Department of Animal Science, Federal University of Pará, Castanhal, Pará, Brazil; 2 Department of Animal Science, Federal Rural University of Amazonia, Belém, Pará, Brazil; 3 Zootechnics Department, Federal Institute of Amapá, Macapá, Amapá, Brazil; Universidade Federal de Mato Grosso do Sul, BRAZIL

## Abstract

Genetic group, age at entry into confinement and at slaughter, are characteristics that have an important influence on lamb performance and carcass. The aim of this study was to evaluate the performance, carcass characteristics and non-carcass components from different genetic groups (Santa Inês and ½ Dorper x ½ Santa Inês) sheep, submitted to different feedlot entry and exit strategies. Were used 72 lambs males and castrated; 36 Santa Inês (SI) and 36 crossbred (Dorper x Santa Inês–DSI), with 6 months of average initial age. The groups were established in a completely randomized experimental design, in a 2x3x4 factorial arrangement, from the combination of genetic groups (GG), body weight at the beginning of confinement (WBC) and length of stay in confinement (LSC). The body weight classes at the beginning of confinement were: light (25 kg), intermediate (28 kg) and heavy (31 kg), for Santa Inês and crossbreeds, respectively. Slaughters were carried out every 28 days of confinement, in four LSC: 0, 28, 56 and 84 days. The GG did not influence performance, carcass and non-carcass component traits of lambs (p > 0.05). There was an effect of the WBC on the weights: final (FW), metabolic (MW), body at slaughter (BWS), empty body (EBW), hot carcass (HCY) and cold (CCW), loin, shoulder, leg musculature; loin eye area (LEA) and loin fat (p < 0.05). There was also an effect on LSC, for FW, average daily weight gain (ADG), MW, weight and yield of body components, weight of cuts and tissue ratio components of cuts (p < 0.05). In non-carcass components, effect on full and empty weight of: omasum, rumen-reticulum, small intestine; empty large intestine, liver and kidneys, paws and skin, and perirenal, pelvic and inguinal fat (p < 0.05). Interaction double effect on the tissue muscle/fat:bone ratio (MF:B) and for the full omasal component (p < 0.05). And triple interaction effect for ADG, full omasum and perirenal fat (p < 0.05). Weight at the beginning of confinement and confinement time are the characteristics that most influence performance, quantitative characteristics of carcass and non-carcass components. Regardless of the genetic group and age class, the animals reach the same weight after 84 days of confinement. Thus, the confinement of heavier lambs (31 kg) can be a profitable alternative, as they presented the highest weights for the most commercially valued cuts (shank and loin). The confinement strategy must adapt to market situations.

## Introduction

The increase in consumption and demand for lamb meat has stimulated research in search of strategies that can help producers in decision-making and increase herds productivity [1, 2]. In this sense, the intensive production system has been fundamental, as it allows for an increase in the stocking rate, regularity of supply and production of better quality products for the consumer market, given that it allows the slaughter of animals in an earlier and more orderly manner [3, 4].

However, even in confinement, the intended responses depend on the individual genetic material, still linked to race or genetic group [5]. Every sheep breed has advantages and disadvantages in meat production traits, and none of them stands out in all desirable traits, ranging from adaptation, precocity, to conformation and carcass quality [6, 7]. Thus, crossings also come in as an alternative, in order to increase the efficiency of the production system [8].

The Santa Inês breed, for example, is known for its maternal ability, hardiness and production of low-fat meat, essential characteristics that have led to crossbreeding with breeds specialized in meat production, such as the Dorper breed, with the possibility of increasing the productive efficiency of the herd [9, 10]. However, the Santa Inês breed, in its place of origin, a highly challenging environment, may have a similar performance in relation to crossbreeding, since the energy expenditure with adaptation may outweigh the benefit of heterosis [11, 12].

In addition, the age and weight of lambs at slaughter are among the main factors that affect meat quality [13, 14], as they influence the characteristics of a good carcass, for example: bone, muscle and fat weight and fat cover. This information are being obtained so that the industry can obtain, for each breed, the ideal slaughter weight, according to consumer requirements [15]. Another important factor is the non-carcass components, which influence carcass yield and, in sheep, are most often discarded without making a profit [16, 17].

Our hypothesis is that genetics, age at slaughter and confinement time can influence performance and carcass yield in lambs. Thus, the objective was to evaluate the performance, carcass characteristics and non-carcass components of Santa Inês and ½ Dorper x ½ Santa Inês sheep, submitted to different feedlot entry and exit strategies.

## Materials and methods

The procedures with the animals were approved by the Ethics Committee for Research with Animals and Experimentation (CEPAE protocol n° 97.2015) of the Federal University of Pará.

### Location, animals and experimental design

The research was conducted in Castanhal, Pará, Brazil (1°17’S and 47°55’W), climate type Am, according to Köppen, average annual temperature of 26.8°C and relative humidity around 85%.

Seventy-two castrated male lambs were used; 36 Santa Inês (SI) and 36 mestizos (½ Dorper x ½ Santa Inês–DSI), with an average initial age of 6 months. The experimental groups were formed based on the combination of genetic groups (GG), body weight at the beginning of confinement (WBC) and length of stay in confinement (LSC). Three body weight classes were formed at the beginning of confinement: Light (25 ± 1.57 and 25 ± 2.40 kg—24 animals), Intermediate (28 ± 1.72 and 28 ± 1.40 kg—24 animals) and Heavy (31 ± 1.01 and 31 ± 1.54 kg—24 animals), for Santa Inês and mestizos, respectively. Slaughters were performed every 28 days, setting up four LSC (days): 0 (6 animals); 28 (6 animals) 56 (6 animals) and 84 (6 animals).

The animals were weighed, dewormed and identified; and confined in individual wooden stalls (1.2 m x 1.0 m), with a concrete floor lined with thick sawdust litter, provided with a feeder and drinker, located in a masonry shed with an opening for natural ventilation.

### Diets

Before confinement, the animals were raised in a continuous grazing system on the grass *Urochloa humidicula* (Quicuio-da-Amazônia), with supplementation of 0.3% of live weight (50% ground corn grain—50% soybean meal) and mineral salt ad libitum. All animals underwent an adaptation period (15 days) to the experimental conditions (environment, management and diets), during which they were weighed, identified, dewormed and vaccinated.

The diet was offered twice a day (8 am and 5 pm) with a ratio of 40:60 (Forage:Concentrate). Was composed of 32% elephant grass (*Pennisetum purpureum*) silage (SIL), 16.30% soybean meal (*Glycine max L*.) (SM), 49.32% ground corn grain (*Zea mays L*.) (CG), 1.03% calcitic limestone (CL) and 1.35% mineral and vitamin supplement (SUP). With 2.31 of Metabolizable Energy (ME) (Mcal/kg diet), 84.4% of Dry Matter (DM), 12.53% of Crude Protein (CP), 1.92% of Ether Extract (EE), 62% of Neutral Detergent Fiber (NDF), 42.63% of Fiber in Acid Detergent (ADF) (Table 1).

**Table 1:** Characterization of the experimental diet.

Ingredients					
%	SIL	SM	CG	CL	SUP
	32.0	16.3	49.32	1.03	1.35
Nutrients					
%	DM	CP	NDF	EE	ME
	84.4	12.53	62	2	2.31

Diet calculated according to NRC recommendations (2007) to meet requirements and gain 200 g/day. SIL = Silage; SM = soybean meal; CG = corn grain; CL = calcitic limestone; SUP = vitamin supplement; DM = Dry Matter; CP = Crude Protein; NDF = Neutral Detergent Fiber; EE = Ether Extract; ME = Metabolizable Energy.

The DM, CP, EE, NDF and ADF were determined according to the Association of Official Analysis Chemists [18]. For estimating Metabolizable Energy (ME) it was assumed that 1 kg of total digestible nutrient (TDN) is equivalent to 4.4 Mcal of digestible energy and multiplied by 0.82 to obtain ME intakes [19]. Feed supply was adjusted daily to ensure 10% leftovers, which were also weighed to determine dry matter intake (DMI).

### Performance

The productive performance of the lambs was evaluated by weighing the animals individually on the first experimental day and on the last day (which varied according to the treatments), always in the morning, before providing the first meal.

Thus, total weight gain (TWG) was determined by the difference between final and initial body weight; and the average daily gain (ADG) dividing the TWG by the number of confinement days (TWG/70). With total daily dry matter intake (total DMI) and average daily gain (ADG), metabolic weight (MW:BW0.75), feed conversion (FC: DMI/ADG ratio) and feed efficiency (FE) was calculated by the following formula: FE = total ADG/DMI. Where: total DMI = total daily dry matter intake; ADG = daily weight gain, in kg/day; and FE = feed efficiency.

### Slaughter

The lambs were slaughtered in an experimental slaughterhouse, following the Regulation of the Industrial and Sanitary Inspection of Products of Animal Origin—RIISPOA [20]. Body weight at slaughter (BWS) and hot carcass weight (HCW) were recorded, used to calculate the hot carcass yield (WCY = 100 * WCW/BWS). The lambs were stunned by electronarcosis, followed by bleeding (section of the carotid arteries and jugular veins), skinning and evisceration, with separation of non-carcass components (NCC): white viscera (rumen-reticulum, omasum and abomasum) and fat deposits (perirenal, pelvic and inguinal), weighed separately. After bleeding and skinning, the carcasses were eviscerated and the non-carcass components were separated into rumen–reticulum, omasum–abomasum, small and large intestines, heart, liver, kidneys, lungs–trachea–esophagus, tongue, blood, and external body components (head, feet, and skin). The gastrointestinal tract (GIT) was initially weighed full. Then it was emptied, washed and weighed again to determine the GIT content and calculate empty body weight. The empty body weight (EBW) was obtained by the difference between slaughter weight and gastrointestinal content.

After 24 hours in a cold room (4°C), the carcasses were weighed, obtaining the cold carcass weight (CCW), used to calculate the cold carcass yield (CCY = 100 * CCW/BWS). Rib eye area (REA) and subcutaneous fat thickness (SFT) were also evaluated [21]. The cooled carcasses were longitudinally divided into two half carcasses, with the right half carcass being used to obtain the commercial cuts (rib, shoulder, loin, leg and neck) [22], weighed separately; and for dissection, in order to estimate the proportions of muscle, bone and adipose tissue in the carcass.

The percentage of muscle, bone and fat (subcutaneous and intermuscular) was calculated by dividing the total of each tissue by the corrected cold carcass weight, then multiplied by 100. The relationships between the various tissues were calculated by dividing the absolute values totals of each component by the other: muscle/bone ratio (M:B) = Total weight of muscles in the carcass/total weight of bones in the carcass; muscle/fat ratio (M:F) = total weight of muscles in the carcass/total weight of fat in the carcass; fat/bone ratio (F:B) = total carcass fat weight/total bone weight in the carcass. Percentages and ratios were found following the methods of [22, 23].

The ratio of the total edible portion to total bones was obtained by adding the total weight of carcass fat and muscle and dividing by the total bone weight: muscle + fat/bone ratio (MF:B) = (Total muscle + fat weight in the carcass) / (total weight of bones in the carcass).

### Statistical analysis

Statistical analyzes were performed in the caret package of the R version 3.5.1 software (R Core Team, 2018). The experimental design was completely randomized in a 2 x 3 x 4 factorial arrangement, considering two genetic groups (SI x DSI), three body weights at the beginning of confinement (25 x 28 x 31 kg) and four times of confinement permanence (0 x 28 x 56 x 84 days).

The normality of data for each variable was verified using the Shapiro-Wilk test [24] and the homogeneity of variances was verified using the Bartlett test after adjusting the model. The variables that did not present normality (full abomasum, full and empty omasum, full rumen-reticulum and perirenal fat) were submitted to the transformation suggested by the Box-Cox procedure [25]. The significance of differences between genetic groups (GG), body weight at the beginning of confinement (WIC), length of stay in confinement (LSC) and interactions were verified by Tukey’s tests, at a significance level of 5%.

## Results

### Performance

The GG did not influence (p > 0.05) performance, carcass traits and non-carcass components of lambs. The WIC was significant (p ≤ 0.05) on the FW and MW variables. The highest averages for both FW (39.74 ± 4.35 kg) and MW (15.81 ± 1.30 kg) were obtained by the heaviest class. For these two variables, there was an effect (p ≤ 0.05) of TCP, with greater weights obtained at 84 days (FW = 40.91 ± 3.21 kg and MW = 16.17 ± 0.95 kg) (Table 2).

**Table 2:** Means ± standard deviations of performance of lambs in different weight classes at the beginning of confinement (WBC, kg) and length of stay in confinement (LSC/days).

Treatment	Variable			
	FW	DMI	ADG	MW
WBC (kg)^(1)^				
25	33.57±5.13 b	1.37±0.31 a	138.33±0.03 a	13.91±1.59 c
28	35.51±4.73 b	1.39±0.53 a	128.06±0.03 a	14.52±1.46 b
31	39.74±4.35 a	1.44±0.54 a	141.74±0.04 a	15.81±1.30 a
Test F	3.50*	2.90^NS^	1.07^NS^	3.68*
LSC (dias)^(2)^				
28	31.36±3.53 c	1.36±0.59 a	117.22±0.03 c	13.23±1.11 c
56	36.54±4.19 b	1.54±0.34 a	146.66±0.04 a	14.84±1.28 b
84	40.91±3.21 a	1.29±0.39 a	142.77±0.03 b	16.17±0.95 a
Test F	13.63*	0.11^NS^	6.06*	17.74***
Test F ^(3)^				
GG x WBC	0.68^NS^	1.22 ^NS^	5.10 ^NS^	0.75^NS^
GG x LSC	0.45^NS^	1.44^NS^	1.04^NS^	0.40^NS^
WBC x LSC	0.55^NS^	1.98^NS^	2.95 ^NS^	0.55^NS^
GG x PIC x LSC	1.22^NS^	2.02^NS^	3.02*	1.19^NS^
CV (%)	6.55	28.94	21.16	4.6

FW: final weight; DMI: Dry matter intake (kg); ADG: Average daily weight gain (g); MW: Metabolic weight (kg); WBC: weight body classes ***(P<0.001), **(P<0.01), *(P<0.05). Means followed by different letters differ by the Tukey test (P<0.05). The values (1, 2 and 3) correspond to the F test statistics, for the individual effects of WBC (1) and LSC (2) and interactions between the factors (3).

There was a triple interaction effect (p ≤ 0.05) between the GG, WIC and TCP factors for the TWG (Table 2), with similar mean gains (p > 0.05) between the GG, in the different WIC classes within each TCP (Table 3), except for the 56 days of confinement, in which the light class SI lambs showed lower gains (103.33 ± 0.01 g) in relation to the heavy class (203.33 ± 0.01 g).

**Table 3:** Means ± standard error of average daily weight gain (ADG/ g) as a function of genetic groups (GG) Santa Inês (SI) and Dorper x Santa Inês (DSI).

TWG		LSC		
GG	WBC	28	56	84
	25	120.00±0.01 Aa	103.33±0.01 Ba	153.33±0.01 Aa
SI	28	66.67±0.01 Aa	146.67±0.01 ABa	130.00±0.01 Aa
	31	146.67±0.01 Aa	203.33±0.01 Aa	113.33±0.01 Aa
	25	136.67±0.01 Aa	146.67±0.01 ABa	170.00±0.01 Aa
DSI	28	126.67±0.01 Aa	153.33±0.01 ABa	143.33±0.01 Aa
	31	106.67±0.01 Aa	126.67±0.01 ABa	147.62±0.01 Aa

TWG: total weight gain; LSC: length of stay in confinement; WBC: weight body classes; Means followed by different uppercase letters in the column and lowercase in the row differ (P < 0.05) from each other by the Tukey test.

### Carcass characteristic

The REA and SFT values were affected by TCP, increasing as the confinement time increased, except for REA, where there was no difference between days 28, 56 and 84 (Table 4). There was a WBC effect for BWS, EBW, HCW and CCW, with higher values in the heavy class WBC (p ≤ 0.05).

**Table 4:** Means ± standard deviations of weights (kg) and yields (%) of body components of lambs in different weight classes at the beginning of confinement (WBC/ kg) and time spent in confinement (LSC/days).

Treatment	Variable							
	BWS	EBW	HCW	HCY	CCW	CCY	REA	SFT
WBC (kg) ^(1)^								
25	30.31±6.12 c	25.93±6.00 c	15.01±4.12 b	48.86±4.50 a	14.01±3.93 b	47.25±5.06 a	12.82±3.17 b	2.58±1.90 a
28	32.07±4.65 b	27.57±4.69 b	16.00± 3.25b	49.55±3.65 a	15.00±3.06 b	47.89±4.11a	13.24±2.41 b	2.68±1.67 a
31	36.20±5.30 a	31.27±5.29 a	18.48±3.53 a	50.78±3.54 a	17.21±3.47 a	49.38±3.91 a	14.83±2.71 a	2.77±1.58 a
Test F	5.65**	4.46*	4.96*	1.34^NS^	4.08*	1.31^NS^	0.60*	0.07^NS^
LSC (days) ^(2)^								
0	26.29±3.11 d	21.46±3.12 d	12.20±1.98 d	46.21±3.37 c	11.12±1.85 d	43.56±3.52 c	11.05± 1.70 b	1.00±0.54 d
28	30.67±3.70 c	26.47±3.40 c	15.00±2.07 c	48.84±2.28 b	14.22±2.07 c	47.75±2.31 b	13.82±2.91 a	2.00±0.75 c
56	35.74±3.87 b	30.95±2.53 b	17.79±2.56 b	49.63±2.96 b	16.77±2.35 b	48.29±3.30 b	14.62±2.74 a	3.10±1.20 b
84	38.75±3.03 a	34.15±2.63 a	21.01±1.75 a	54.23±2.05 a	19.53±3.94 a	53.08±2.15 a	15.04±2.34 a	4.60±1.56 a
Test F	26.40***	25.96***	26.36***	4.26**	25.00***	5.70**	4.80**	15.76***
Test F ^(3)^								
GG x WBC	0.02^NS^	0.17^NS^	0.04^NS^	0.48^NS^	0.02^NS^	0.54^NS^	0.26^NS^	0.20^NS^
GG x LSC	0.63^NS^	0.43^NS^	0.57^NS^	0.59^NS^	0.42^NS^	0.25^NS^	0.92^NS^	2.80^NS^
WBC x LSC	0.67^NS^	0.58^NS^	0.58^NS^	1.05^NS^	0.90^NS^	0.59^NS^	0.64^NS^	1.60^NS^
GG x WBC x LSC	0.62^NS^	0.55^NS^	0.90^NS^	1.24^NS^	0.87^NS^	1.08^NS^	0.34^NS^	1.72^NS^
CV (%)	6.98	7.89	8.85	5.24	9.47	5.77	16.50	36.22

BWS: Body weight at slaughter (kg); EBW: Empty body weight (kg); WCW: Hot carcass weight (kg); HCY: Hot carcass yield (%); CCW: Cold carcass weight (kg); CCY: Cold carcass yield (%); REA: loin eye area (cm^2^); SFT: Subcutaneous fat thickness (mm)

***(P<0.001)

**(P<0.01)

*(P<0.05).

Means followed by distinct letters differ (P<0.05) from each other by the Tukey test. The values (1, 2 and 3) correspond to the F test statistics, for the individual effects of WBC (1) and LSC (2) and interactions between the factors (3).

Higher loin and shoulder weights were obtained (p < 0.05) by the heaviest confined animals, with mean weights of 1.13 ± 0.21 kg and 3.0 ± 0.53 kg, respectively (Table 5). Regarding the effect of TCP on the weights of the cuts, the highest weights were observed at 84 days, except for the neck, which presented similar results at 28, 56 and 84 days (p ≤ 0.05). There was no interaction effect (p > 0.05) for the cuts studied.

**Table 5:** Means ± standard deviations of weights (kg) of commercial cuts of lambs in different classes of weight at the beginning of confinement (WBC/kg) and time spent in confinement (LSC/days).

Treatment	Sirloin	Palette	Neck	Shank	Ribs
WBC (kg)^(1)^					
25	0.91±0.28 c	2.44±0.56 c	0.85±0.21 a	4.65±1.14 a	5.10±1.79 a
28	1.01±0.23 b	2.70±0.53 b	0.84±0.17 a	4.94±0.88 a	5.57±1.49 a
31	1.13±0.21 a	3.00±0.53 a	1.00±0.22 a	5.64±1.06 a	6.43±1.64 a
Test F	6.70**	3.25*	0.61^NS^	3.13^NS^	2.65^NS^
LSC (days)^(2)^					
0	0.80±0.18 c	2.05±0.31 d	0.68±0.10 b	3.79±0.58 d	3.79±0.81 d
28	0.88±0.18 c	2.53±0.35 c	0.90±0.16 a	4.81±0.63 c	5.09±0.93 c
56	1.11±0.20 b	2.92±0.35 c	0.99±0.17 a	5.50±0.73 b	6.24±1.12 b
84	1.27±0.14 a	3.33±0.32 a	1.02±0.21 a	6.21±0.63 a	7.68±0.89 a
Test F	15.31***	12.23***	4.31**	18.90***	21.83***
Test F ^(3)^					
GG x WBC	1.32^NS^	0.006^NS^	0.18^NS^	0.04^NS^	0.05^NS^
GG x LSC	1.35^NS^	0.20^NS^	1.05^NS^	0.36^NS^	0.49 ^NS^
WBC x LSC	1.17^NS^	0.31^NS^	0.90^NS^	0.57^NS^	1.48^NS^
GG x WBC x LSC	0.97^NS^	0.38^NS^	0.91^NS^	0.74^NS^	1.10^NS^
CV (%)	13.29	9.88	54.83	9.70	12.69

GG: Genetic cluster; PIC: Body weight at the beginning of confinement; LSC: length of stay in confinement

***(P<0.001)

**(P<0.01)

*(P<0.05). Means followed by different letters differ by the Tukey test (P<0.05). The values (1, 2 and 3) correspond to the F test statistics, for the individual effects of WBC (1) and LSC (2) and interactions between the factors (3).

Only LSC was significant (p ≤ 0.05) on carcass tissue components and their relationships (Table 6), with higher means at 84 days, except for M:F. For this tissue relationship, the largest occurred in the initial LSC (0 to 28 days) with mean weights of 3.68 ± 1.11 and 3.20 ± 0.60 kg, respectively. In addition, there was an effect of the interaction between GG and LSC for MF:B (Table 6), with an increase in the weight of the edible portion over time up to the 56 days of confinement (p ≤ 0.05) (Table 7). From then on, there was no increase in the edible portion until 84 days of confinement (p > 0.05).

**Table 6:** Means ± standard deviations of muscle, bone and adipose tissue ratio (kg) of lambs.

MF:B	LSC			
GG	0	28	56	84
SI	2.60±0.20 Ac	3.92±0.18 Ab	4.16±0.18Aab	4.88±0.18Aa
DSI	3.23±0.20 Ab	3.72±0.18 Ab	4.62±0.18 Aa	5.11±0.18 Aa

GG: Genetic cluster; WBC: Body weight at the beginning of confinement; LSC: Length of stay in confinement; ***(P < 0.001), **(P < 0.01), *(P < 0.05). Means followed by different letters differ by the Tukey test (P<0.05). The values (1, 2 and 3) correspond to the F test statistics, for the individual effects of WBC (1) and LSC (2) and interactions between the factors (3).

**Table 7:** Means ± standard error of muscle + fat:bone ratio (MF:B, kg) as a function of genetic grouping (GG) and length of stay in confinement (LSC/days).

Variable											
Treatment	Palette			Shank			Sirloin Ribs				
	TM	TF	TB	TM	TF	TB	TM	TF	TM	TF	TB
WBC (kg)^(1)^											
25	1.47±0.35 a	0.38±0.20 a	0.54±0.11 a	3.02±0.79 b	0.65±0.27 a	0.87±0.14 a	0.47±0.17 c	0.17±0.10 b	2.31±0.67 a	1.71±1.01 a	0.92±0.18 a
28	1.59±0.36 a	0.41± 0.19 a	0.58±0.08 a	3.16±0.60 b	0.67±0.28 a	0.89±0.10 a	0.56±0.14 b	0.19±0.10 ab	2.50±0.58 a	1.83±0.87 a	0.97±0.13 a
31	1.78±0.35 a	0.52 ±0.21 a	0.62±0.09 a	3.63±0.71 a	0.84±0.29 a	0.94±0.12 a	0.65± 0.15 a	0.23±0.08 a	2.84±0.70 a	2.23±0.96 a	1.14±0.20 a
Test F	1.06^NS^	0.37^NS^	2.10^NS^	3.48*	0.68^NS^	2.50^NS^	4.47*	5.63**	1.91^NS^	1.10^NS^	0.92^NS^
LSC (days)^(2)^											
0	1.14±0.19 d	0.21±0.07 d	0.51±0.11 b	2.30±0.46 d	0.41±0.13 d	0.81±0.12 c	0.38±0.10 c	0.12±0.06 c	1.77±0.34 d	0.93±0.41 d	0.94±0.19 b
28	1.54±0.16 c	0.34±0.11 c	0.53±0.08 b	3.18±0.37 c	0.59±0.21 c	0.85±0.09 bc	0.53± 0.10 b	0.14±0.06 c	2.29±0.36 c	1.38±0.47 c	0.96±0.21 b
56	1.72±0.21 b	0.51±0.17 b	0.62±0.06 a	3.52±0.46 b	0.83±0.19 b	0.92±0.11 ab	0.61±0.16 b	0.22±0.07 b	2.75±0.44 b	2.16±0.62 b	1.01±0.13 ab
84	2.00±0.23 a	0.65±0.14 a	0.65±0.07 a	3.98±0.44 a	1.02±0.17 a	0.99±0.09 a	0.72±0.10 a	0.31±0.06 a	3.30±0.42 a	3.10±0.49 a	1.13±0.18 a
Test F	11.56***	6.70***	4.32**	16.75***	10.16***	6.55***	14.75***	12.71***	13.96***	17.66***	3.90*
Test F ^(3)^											
GG x WBC	0.39^NS^	0.02^NS^	0.74^NS^	0.88^NS^	0.09^NS^	1.03^NS^	2.18^NS^	1.03^NS^	0.09^NS^	0.12^NS^	0.59^NS^
GG x LSC	0.61^NS^	0.21^NS^	0.33^NS^	0.20^NS^	0.85^NS^	1.00^NS^	1.41^NS^	0.13^NS^	0.21^NS^	0.40^NS^	0.32^NS^
WBC x LSC	0.66^NS^	0.26^NS^	1.23^NS^	0.67^NS^	0.31^NS^	1.76^NS^	2.08 ^NS^	2.20^NS^	0.83^NS^	1.05^NS^	1.69^NS^
GG x WBC x LSC	0.30^NS^	0.48^NS^	0.88^NS^	0.84^NS^	0.37^NS^	1.38^NS^	1.30^NS^	0.96^NS^	0.51^NS^	1.94^NS^	1.29^NS^
CV (%)	10.78	26.98	13.12	10.73	23.14	11.03	14.77	28.56	12.91	20.65	15.01

WBC: weight body classes; Means followed by different uppercase letters in the column and lowercase in the row differ by the Tukey test (P < 0.05).

#### Non-carcass components

In the tissue composition of the cuts and ratios, there was an effect of WBC on the leg and loin musculature, with higher averages for the heavy class, with an average weight of 3.63 ± 0.71 and 0.65 ± 0.15 kg, respectively (p ≤ 0.05) (Table 8); and loin fat, with higher weights in the intermediate (0.19 ± 0.10 kg) and heavy (0.23 ± 0.08 kg) classes, but there was no difference between them. All these variables had a significant effect on LSC (p ≤ 0.05), with higher weights at 84 days, except for the bone weight of the sirloin cut which were similar.

**Table 8:** Means ± standard deviations of muscle, bone and adipose tissue ratio of tissue components (kg) of lambs.

Variable										
Treatment	Full abomasum	Empty abomasum	Full omasum	Empty omasum	Full rumen-reticulum	Empty rumen-reticulum	Full small intestine	Empty small intestine	Full large intestine	Empty large intestine
LSC (days)^(1)^										
0	0.30±0.13 a	0.13±0.04 a	0.22±0.05 a	0.09±0.01 a	4.29±0.81 a	0.66 ±0.10b	0.81±0.12 b	0.56±0.09ab	0.96±0.18 a	0.42±0.10 a
28	0.37±0.12 a	0.14±0.03 a	0.15±0.04 b	0.07±0.01 b	3.55±0.51 a	0.74±0.16 ab	0.90±0.20 ab	0.45±0.13 b	0.99±0.19 a	0.32±0.05 b
56	0.32±0.10 a	0.15±0.03 a	0.16±0.03 b	0.07±0.01 b	3.96±0.58 a	0.76±0.08 a	1.03±0.19 a	0.60±0.14 a	1.03±0.27 a	0.38±0.09 ab
84	0.33±0.15 a	0.15±0.04 a	0.16±0.03 b	0.07±0.01 b	4.07±0.67 a	0.80±0.11 a	0.91±0.15 ab	0.52±0.09 ab	1.04±0.17 a	0.34±0.05 b
Test F	1.52^NS^	2.14^NS^	1.28*	3.67*	0.91 ^NS^	5.21*	1.95*	0.41*	0.52^NS^	0.13*
Test F^(2)^										
GG x WBC	0.77^NS^	0.27^NS^	0.01^NS^	1.63^NS^	1.35^NS^	0.79^NS^	0.20^NS^	0.93^NS^	0.05^NS^	0.43^NS^
GG x LSC	1.06^NS^	0.67^NS^	0.50^NS^	0.24^NS^	0.41^NS^	1.27^NS^	0.01^NS^	0.71^NS^	1.65^NS^	0.23^NS^
WBC x LSC	0.93^NS^	0.64^NS^	1.10**	1.10^NS^	0.84^NS^	1.23 ^NS^	1.40^NS^	1.23^NS^	0.02^NS^	1.36^NS^
GG x WBC x LSC	0.32^NS^	0.61^NS^	1.97***	2.17 ^NS^	0.70^NS^	2.55 ^NS^	1.65^NS^	1.26^NS^	0.84^NS^	0.48^NS^
CV (%)	44.23	26	19.12	19.8	14.78	14.37	18.72	22.56	56.12	21.11

LSC: length of stay in confinement; GG: genetic groups; WBC: weight body classes; Means followed by different letters differ by the Tukey test (P<0.05).

***(P < 0.001)

**(P < 0.01)

*(P < 0.05). Values (1 and 2) correspond to the F test statistics, for the individual effects of LSC (1) and interactions between factors (2).

In the components of the gastrointestinal tract, LSC influenced the empty rumen-reticulum, with lower weight at the beginning of confinement (0.81 ± 0.10 kg) and increase in the other LSC, but there was no difference between them (p ≤ 0.05) (Table 9). The same behavior was observed in the full small intestine, with greater weight at the beginning of confinement (0.81 ± 0.12 kg). The empty omasal component had greater weight on day 0 (0.09 ± 0.01 kg) and exactly the same in the other LSC (0.07 ± 0.01 kg). There was also an effect of the interaction between WBC and LSC and between GG, WBC and LSC for the full omasal component (p ≤ 0.05) (Table 9). For the double interaction, the heavy class stood out, which obtained greater weight for the full omasal component at the beginning of confinement, with no differences between the other times (28, 56 and 84) (Table 10).

**Table 9:** Means ± standard deviations of the weights of the components of the gastrointestinal tract (kg) of the lambs.

Variables											
Treatments	Liver	Kidneys	Heart	Spleen	Lungs/Trachea	Paws	Tongue	Skin	Fats		
									Perirenal	Pelvic	Inguinal
LSC (dias)											
0	0,37±0,06 c	0,10±0,01 a	0,12±0,02 a	0,08±0,03 a	0,47±0,09 a	0,72±0,09 c	0,07±0,00 a	2,15±0,37 b	0.05±0.02 c	0.01±0.01 c	0.008±0.07 c
28	0,49±0,08 b	0,08±0,01 b	0,12±0,02 a	0,07±0,02 a	0,41±0,12 a	0,80±0,10 b	0,08±0,02 a	2,38±0,48 b	0.09±0.03 c	0.05±0.02 b	0.01±0.00 bc
56	0,54±0,06 ab	0,10±0,01 a	0,14±0,01 a	0,08±0,01 a	0,46±0,07 a	0,91±0,07 a	0,08±0,01 a	2,76±0,40 a	0.14±0.06 b	0.02±0.01 c	0.08±0.03 a
84	0,57±0,07 a	0,09±0,01 a	0,14±0,02 a	0,08±0,02 a	0,49±0,08 a	0,95±0,11 a	0,09±0,00 a	2,80±0,35 a	0.22±0.07 a	0.12±0.03 a	0.03±0.01 b
Test F	10,20***	4,17*	2,18^NS^	0,36^NS^	0,36^NS^	7,44***	1,60^NS^	2,8*	11.60***	26.50***	11.05***
Test F											
GG x WBC	0,09^NS^	1,55^NS^	0,19^NS^	0,19^NS^	0,05^NS^	0,15 ^NS^	0,02^NS^	0,38^NS^	0.00^NS^	0.01^NS^	0.05^NS^
GG x LSC	0,67^NS^	0,62^NS^	0,25^NS^	0,78^NS^	1,00^NS^	0,61 ^NS^	1,17^NS^	0,13^NS^	1.88^NS^	0.45^NS^	1.65^NS^
WBC x LSC	0,85^NS^	0,74^NS^	0,97^NS^	0,62^NS^	1,99^NS^	1,52 ^NS^	1,26^NS^	0,33^NS^	1.89^NS^	1.50^NS^	0.02^NS^
GG x WBC x LSC	1,40^NS^	2,13^NS^	0,28^NS^	0,81^NS^	3,32^NS^	1,21 ^NS^	1,22^NS^	1,11^NS^	2.39*	1.67^NS^	0.84^NS^
CV (%)	13,11	11,43	15,02	33,31	18,93	10,50	16,63	13,94	37.38	36.11	56.12

LSC: length of stay in confinement; GG: genetic groups; WBC: weight body classes; Means followed by different letters differ by the Tukey test (P<0.05).

***(P < 0.001)

**(P < 0.01)

*(P < 0.05). Values (1 and 2) correspond to the F test statistics, for the individual effects of LSC (1) and interactions between factors (2).

**Table 10:** Means ± standard error of the full omasal component (kg) as a function of different weights at the beginning of confinement (WBC/kg) and length of stay in confinement (LSC/days).

Full omasum	LSC			
WBC	0	28	56	84
25	0.19±0.01Aa	0.13±0.01 Aa	0.16±0.01 Aa	0.15±0.02 Aa
28	0.22±0.01 Aa	0.17±0.01 Aa	0.15±0.01 Aa	0.15±0.02 Aa
31	0.25±0.01 Aa	0.15±0.01 Ab	0.17±0.01 Ab	0.17±0.02 Ab

Means followed by different uppercase letters in the column and lowercase in the row differ by the Tukey test (P < 0.05).

Mean full omasal weights were similar for GG across different WBC classes within each LSC (p > 0.05) (Table 11). Only in the heavy class (SI), there was a difference in the weight of the full omasum between the beginning of confinement and the 56th day (p ≤ 0.05).

**Table 11:** Means ± standard error of the full omasal component (g) as a function of genetic groups (GG) and different weight classes at the beginning of confinement (WBR, kg) and length of stay in confinement (LSC, days).

Full omasum		LSC			
GG	WBC	0	28	56	84
	25	0.20±0.018 Aa	0.13±0.018 Aa	0.16±0.018 Aa	0.17±0.018 Aa
SI	28	0.23±0.018 Aa	0.16±0.018 Aa	0.16±0.018 Aa	0.17±0.018 Aa
	31	0.28±0.018 Aa	0.19±0.018 Aab	0.14±0.018 Ab	0.16±0.018 Aab
	25	0.18±0.018 Aa	0.14±0.018 Aa	0.16±0.018 Aa	0.13±0.018 Aa
DSI	28	0.21±0.018Aa	0.19±0.018 Aa	0.13±0.018 Aa	0.13±0.018 Aa
	31	0.23±0.018 Aa	0.10±0.018 Aa	0.19±0.018 Aa	0.18±0.018 Aa

Means followed by different uppercase letters in the column and lowercase in the row differ by the Tukey test (P < 0.05).

The LSC was the factor that most influenced the non-carcass components (Table 12). In perirenal and pelvic fat, the highest weights occurred at 84 days 0.22 ± 0.07 kg and 0.12 ± 0.03 kg, respectively. Likewise, for the liver, paws and skin component, there was an increase in weights from 56 days of confinement and similar to 84 days. For kidneys, the lowest weight occurred with 28 days of confinement (0.08 ± 0.01 kg) and exactly the same for the other LSC.

**Table 12:** Means ± standard deviations of non-carcass component weights (kg) of lambs.

Variables											
	Liver	Kidneys	Heart	Spleen	Lungs/Trachea	Paws	Tongue	Skin	Fats		
Treatment									Perirenal	Pelvic	Inguinal
LSC (days)											
0	0,37±0,06 c	0,10±0,01 a	0,12±0,02 a	0,08±0,03 a	0,47±0,09 a	0,72±0,09 c	0,07±0,00 a	2,15±0,37 b	0.05±0.02 c	0.01±0.01 c	0.008±0.07 c
28	0,49±0,08 b	0,08±0,01 b	0,12±0,02 a	0,07±0,02 a	0,41±0,12 a	0,80±0,10 b	0,08±0,02 a	2,38±0,48 b	0.09±0.03 c	0.05±0.02 b	0.01±0.00 bc
56	0,54±0,06 ab	0,10±0,01 a	0,14±0,01 a	0,08±0,01 a	0,46±0,07 a	0,91±0,07 a	0,08±0,01 a	2,76±0,40 a	0.14±0.06 b	0.02±0.01 c	0.08±0.03 a
84	0,57±0,07 a	0,09±0,01 a	0,14±0,02 a	0,08±0,02 a	0,49±0,08 a	0,95±0,11 a	0,09±0,00 a	2,80±0,35 a	0.22±0.07 a	0.12±0.03 a	0.03±0.01 b
Test F	10,20***	4,17*	2,18^NS^	0,36^NS^	0,36^NS^	7,44***	1,60^NS^	2,8*	11.60***	26.50***	11.05***
Test F											
GG x WBC	0,09^NS^	1,55^NS^	0,19^NS^	0,19^NS^	0,05^NS^	0,15 ^NS^	0,02^NS^	0,38^NS^	0.00^NS^	0.01^NS^	0.05^NS^
GG x LSC	0,67^NS^	0,62^NS^	0,25^NS^	0,78^NS^	1,00^NS^	0,61 ^NS^	1,17^NS^	0,13^NS^	1.88^NS^	0.45^NS^	1.65^NS^
WBCx LSC	0,85^NS^	0,74^NS^	0,97^NS^	0,62^NS^	1,99^NS^	1,52 ^NS^	1,26^NS^	0,33^NS^	1.89^NS^	1.50^NS^	0.02^NS^
GG x WBC x LSC	1,40^NS^	2,13^NS^	0,28^NS^	0,81^NS^	3,32^NS^	1,21 ^NS^	1,22^NS^	1,11^NS^	2.39*	1.67^NS^	0.84^NS^
CV (%)	13,11	11,43	15,02	33,31	18,93	10,50	16,63	13,94	37.38	36.11	56.12

LSC: length of stay in confinement; GG: genetic groups; Means followed by different letters differ by the Tukey test (P<0.05)

*(P<0.05)

Values (1 and 2) correspond to the F test statistics, for the individual effects of LSC (1) and interactions between factors (2).

There was an interaction effect (p ≤ 0.05) between GG, WBC and LSC for the lung-trachea component and perirenal fat (Table 11). In perirenal fat, similar weights were observed for GG in different WBC classes, within each LSC (p > 0.05) (Table 13). Only in SI (heavy class) and DSI (light class) lambs, higher weights of this fat were observed in longer confinement times (56 and 84 days). In the light class SI lambs, there was no difference between the means of the perirenal fat component at 28 and 84 days (p > 0.05). For the other WBC classes within the genetic groups, there was no difference between the means of this component over the confinement time (p > 0.05).

**Table 13:** Means ± standard error of perirenal fat (kg) as a function of genetic groups (GG), different weight classes at the beginning of confinement (WBC/kg) and length of stay in confinement (LSC/days).

Fat perirenal				LSC			
GG		WBC	0		28	56	84
SI		25	0.03±0.027 Ab		0.10±0.027 Aab	0.07±0.027 Ab	0.24±0.027 Aa
		28	0.04±0.027 Aa		0.10±0.027 Aa	0.16±0.027 Aa	0.17±0.027 Aa
		31	0.05±0.027 Ab		0.11±0.027 Ab	0.16±0.027 Aab	0.30±0.027 Aa
		25	0.05±0.027 Ab		0.07±0.027 Ab	0.11±0.027 Aab	0.26±0.027 Aa
DSI	28		0.07±0.027 Aa		0.05±0.027 Aa	0.16±0.027 Aa	0.20±0.027 Aa
	31		0.08±0.02 Aa		0.12±0.027 Aa	0.14±0.027 Aa	0.15±0.027 Aa

Means followed by different uppercase letters in the column and lowercase in the row differ by the Tukey test (P < 0.05).

## Discussion

### Performance

The crossbred DSI lambs obtained characteristics similar to those of the Santa Inês breed, data consistent with previous reports [26–28], indicating absence of the effect of heterosis. It is conjectured that the crossbred lambs did not express their genetic potential, given a possible unfavorable environmental condition in their previous commercial breeding systems, causing some restriction in the initial phase of growth, since the animals were submitted to confinement at 6 months.

The DM intakes for the two genetic groups were similar to those found by [29] for the same genotypes with an average time spent in confinement of 36 days; and those found by Saldanha [12]. The intake obtained in this study is in accordance with that recommended by the [19], which vary from 1.05 to 1.32 kg/day for this animal category, ranging from 1.31 to 1.49 kg day-1 for the genetic groups. For the ADG, the interaction between GG, WBC and LSC that the Santa Inês lambs obtained gains of 203.33 g/day for the intermediate class at 56 days of confinement, differing from those presented by [30, 31] with the same genetic groups, in which crossbreeds reached higher weights, but higher than those found by [32].

The fact that FC and EF, indices commonly used to measure nutritional performance, did not show significant effects, indicates that the lambs, regardless of GG and WBC, were similar in the transformation of dry matter into body weight. On the other hand, the justification for the feed conversion not being different for the genetic groups was probably due to the fact that CMS and ADG did not present significant differences for the means of these traits. These results are similar to those found by [26, 33, 34], for the same genetic groups, with similar weights at the beginning of confinement and obtained similar final weights over the confinement time.

### Carcass characteristics and non-carcass components

It was expected that confined animals (heaviest group) would obtain higher carcass, REA and STF yields. On the other hand, there was an increase in non-carcass components, mainly viscera, with the exception of the omasum, a factor that may be directly linked to the proportion of energy supplied in the diets. Highly energetic diets produce a greater deposition of fat in the carcass and viscera, causing the involution of the omasum [35]. Furthermore, the lambs that remained longer in confinement, despite the greater weight gain, also showed an increase in visceral fat.

It should be noted that the weights of the body components are related to the stage of development of the animal, that is, the greater its degree of physiological maturity and body weight, the greater the weight of the cuts, and are also influenced by the supply of a diet concentrated [22, 36, 37].

That said, the heavy class obtained higher values for FW, MW, BWS, EBW, HCW, CCW and commercial cuts loin and shoulder. In addition, they showed an increase in all characteristics of body components, yields and weight of the cuts over the confinement time. Confining with 31 kg also resulted in higher final weight at 84 days, when compared to the weight ranges of 25 and 28 kg. Furthermore, greater weights of the tissue components of the ham muscle and loin fat were observed in this class. It is important to emphasize that the loin and shank are the most commercially valued cuts [27], and the confinement of heavier lambs (31 kg) can be a profitable alternative.

The increase in the weight of the cuts in all periods with the longest confinement time was due to the longer time for the animals to adapt and body development, which favored the weight gain of the meat cuts. The results are in agreement with those obtained by [38], who also obtained a higher proportion for the leg of lambs slaughtered at 60 days old compared to those slaughtered at 40 days old; and [39].

In terms of physiological priority, early development of bone tissue, intermediate development of muscle fibers, and late development of adipose tissue are observed [39, 40]. The muscle develops proportionally to the animal’s body weight, the longer they remain in confinement, decreasing close to the age of sexual maturity, when they reach a certain maximum level of muscle development [37]. On the other hand, when the animal reaches puberty, according to the theoretical curve of animal growth, fat deposition becomes representative, contributing to weight gain and resulting in changes in the individual’s conformation, as well as in body composition (carcass and organs) [40–43].

Crossbred and SI lambs in this study had an average age of 6 months at the beginning of confinement, being considered animals closer to the adult stage, which occurs at 1 year of age in lambs. In this phase, the animals are already finishing muscle deposition and starting the process of adipose tissue deposition [37]. As the animal advances towards maturity, the percentage of fat and the muscle-bone ratio increases; the proportion of muscle in the carcass decreases and the proportion of fat increases as body weight increases, as well as the biochemical composition of the tissues also changes, with the gain of lipids in the muscle and the loss of water, also with the advancement of maturity [14, 44].

Thus, the increase in fat deposition, with the time spent in confinement, influenced the M:F ratio, which suffered a decreasing effect, as the lambs increased their weight, demonstrating that the greater the age at slaughter, the greater the weight, adiposity and carcass conformation [45, 46], with weight and age at slaughter being fundamental factors that determine meat quality and make it possible to increase the adoption of strategies during decision-making that encourage the production of sheep meat and the choice of the best slaughter period according to the current needs of the Brazilian industry [38, 47].

The edible portion increased with the confinement time. Thus, interpreting the existing relationship between the tissue composition (muscle, fat and bone) present in the carcass makes it possible to determine the best period for slaughter, providing the market with carcasses with greater quantity and better distribution of muscularity and adiposity, and meat with better quality sensory, thus obtaining attractive quality carcasses for consumers [48–51].

The variables abomasum (full and empty), rumen-full reticulum and the organs: lung/trachea, heart, spleen and tongue, were not influenced by the factors. Non-carcass components such as: omasum, rumen and reticulum, present late development, unlike the abomasum (early), and the percentages of some non-carcass components decrease with increasing live weight of animals [52]. However, the rumen-reticulum weight reached 0.80 kg, higher than those found by [53], with an average weight of 0.46 kg. The diet with high levels of NDF and ADF probably increased food retention in the rumen and provided greater development of this organ.

Liver and kidneys, and confinement time were the most relevant factors. This fact may have occurred in response to a higher metabolic rate, due to the level of concentrate present in the diet, and it can be inferred that the development of the organs is also linked to the size of the animals, consumption and composition of diet [2, 54], which provided an increase over the length of stay in confinement [39, 55], as well as for paws and skin.

The non-carcass component (omasum full) showed an increase in weight at the beginning of confinement, but it was not effective over time. Organs and viscera develop early and the phenomenon occurs with greater intensity in the early stages of the animal’s life, shortly after weaning and with the beginning of feeding, mainly forage [56, 57]. With the course of the animal’s age, there is some speed growth of muscle tissues, but which reduces in intensity with the arrival of puberty, initiating the increase in fat deposition [58, 59].

Thus, perirenal fat deposits increased with time spent in feedlot for heavy classes (SI) and light class for lambs (DSI), in confirmation of age in relation to the growth of adipose tissue. But, it is necessary to consider the extent to which fat is interesting in the animal’s carcass, as in large amounts it will harm the producer [54]. In some countries, due to the population’s preference for meat with a higher percentage of fat, breeds with this tendency are created, and the slaughter takes place later, however, more is paid for the final product [60–62].

Evaluating different slaughter weights (28, 32, 36 and 40 kg), Siqueira [63] observed that non-carcass constituents, such as the stomach, showed fluctuations in terms of their weight as they remained in feedlot. A high percentage of NDF and ADF provide lower digestibility and, consequently, greater retention of food in the reticulum rumen, thus resulting in greater development of the same [64, 65]. Thus, lambs of different weight classes, as they remain longer in confinement, show less efficiency and greater food retention in the gastrointestinal tract content, promoting an increase in their rumen capacity.

In general, confining lambs with 31 kg implies a higher final weight at 84 days, when compared to the other weight ranges (25 and 28 kg), with higher values for BWS, EBW, HCW, CCW of the cuts and non-carcass components. In the relationship between GG, WBC and LSC, feedlot SI lambs with higher initial weight had higher weight of non-carcass components, indicating that part of the BWS is due to the viscera. In the case of DSI lambs, a lower viscera weight was observed at the end of 84 days, when confined with 31 kg. However, the tissue ratio of the edible portion for the two genetic groups increased over time in confinement, indicating that a longer stay in confinement brings good results.

## Conclusion

The weight at the beginning of confinement and the time spent in confinement are characteristics that most influence performance, quantitative characteristics of the carcass and non-carcass components. Regardless of the genetic group and age class, the animals reach the same weight after 84 days of confinement. Thus, the confinement of heavier lambs (31 kg) can be a profitable alternative, as they presented the highest weights for the most commercially valued cuts (shank and loin). The confinement strategy must adapt to market situations.
